# The circular RNA *Cdr1as*, via miR-7 and its targets, regulates insulin transcription and secretion in islet cells

**DOI:** 10.1038/srep12453

**Published:** 2015-07-27

**Authors:** Huanyu Xu, Sen Guo, Wei Li, Ping Yu

**Affiliations:** 1 Institute of Genomic Medicine, Wenzhou Medical University, Zhejiang, China; 2 Zhejiang Provincial Key Laboratory of Medical Genetics; School of Laboratory Medicine & Life Sciences, Wenzhou Medical University, Zhejiang, China

## Abstract

Among the identified thousands of circular RNAs (circRNA) in humans and animals, *Cdr1as* (also known as *CiRS-7*) was recently demonstrated to act as a powerful miR-7 sponge/inhibitor in developing midbrain of zebrafish, suggesting a novel mechanism for regulating microRNA functions. MiR-7 is abundantly expressed in islet cells, but overexpressing miR-7 in transgenic mouse β cells causes diabetes. Therefore, we infer that *Cdr1as* expression may inhibit miR-7 function in islet cells, which in turn improves insulin secretion. Here, we show the first characterization of *Cdr1as* expression in islet cells, which was upregulated by long-term forskolin and PMA stimulation, but not high glucose, indicating the involvement of cAMP and PKC pathways. Remarkably, both insulin content and secretion were significantly increased by overexpression of *Cdr1as* in islet cells. We further identified a new target *Myrip* in the *Cdr1as*/miR-7 pathway that regulates insulin granule secretion, and also another target *Pax6* that enhances insulin transcription. Taken together, our findings revealed the effects of the strongly interacting pair of *Cdr1as*/miR-7 on insulin secretion, which may become a new target for improving β cell function in diabetes.

The identification and characterization of circular RNA (or circRNA) have recently reshaped the RNA world. CircRNA is a type of RNA that forms a covalently closed continuous loop, which has been detected in viruses^1^, plant^2^, archaea^3^, and animals^4^. Following high-throughput sequencing and bioinformatics analysis, thousands of different circRNAs have been identified in recent several years^5^,^6^,^7^,^8^,^9^,^10^,^11^. Initial characterization showed that circRNAs are abundantly expressed and evolutionarily conserved across the eukaryotic tree of life^7^,^10^. Particularly, many of them are evolutionary conserved between humans and mice^7^.

CircRNAs are predominantly found in cytoplasm, highly stable and untranslated. Most of them come from gene exons, and are thus called exonic circRNAs. CircRNAs could be produced by a non-canonical mode of RNA splicing^5^. Several other studies suggested that circRNA biogenesis is contributed by back-splicing pre-mRNA transcripts and exon-skipping events^12^,^13^,^14^. More recently, exon circularization was found to be correlated with flanking intronic complementary sequences^15^, while this circularization competes with linear splicing in a tissue-specific fashion^16^. Like linear mRNAs, thousands of well-expressed, stable expressions of circRNAs are tissue-specific and/or developmental-stage-specific^7^,^11^.

However, biological functions of circRNAs remain to be elucidated. Due to the abundance and evolutionary conservation, several potential functions of circRNAs have been predicted^17^,^18^ and initially demonstrated to be miRNA sponges^6^,^7^. Until very recently, one of the circRNAs, named as *Cdr1as* (also known as *ciRS-7*), was demonstrated *in vivo* to function as the miR-7 sponge or inhibitor^6^,^7^. Overexpression of *Cdr1as* in embryonic zebrafish brain induced developmental defects in midbrain, which was similar to the phenotypes observed in the miR-7 knockdown zebrafish^7^. This phenotype is attributed to the alteration of miR-7 expression level in central nervous systems (CNS)^19^,^20^. Beyond CNS, miR-7 is also abundantly expressed in adult mouse and human islet cells and plays an important role in inhibiting insulin granule exocytosis^21^,^22^,^23^,^24^. Transgenic mice overexpressing miR-7 in β cells developed diabetes due to reduced insulin secretion and impaired β cell dedifferentiation, whereas that inactivation of miR-7 in obese mice might be sufficient to rescue β cell failure and glycemia by enhancing β cell maturity and pancreatic function^24^. Therefore, it is interesting to investigate whether *Cdr1as*, as a powerful miR-7 inhibitor/sponge, could improve the adaptation of islet cells for insulin secretion and could ultimately serve as a potential treatment target for obese diabetes.

In the current study, we first characterized the *Cdr1as*/miR-7 axis in terms of their expression in β cells and then studied the effects of several secretagogues for activation of insulin signalling on the expressions of *Cdr1as*/miR-7. Furthermore, we explored *Cdr1as*’s function in insulin content and secretion and identified, to the best of our knowledge, a novel effector of the miR-7, which attributed to the insulin granule secretion. Additionally, we discussed a possible model explaining the *Cdr1as*/miR-7 network in islet cells.

## Results

### Characterization of *Cdr1as*

**Figure 1:**
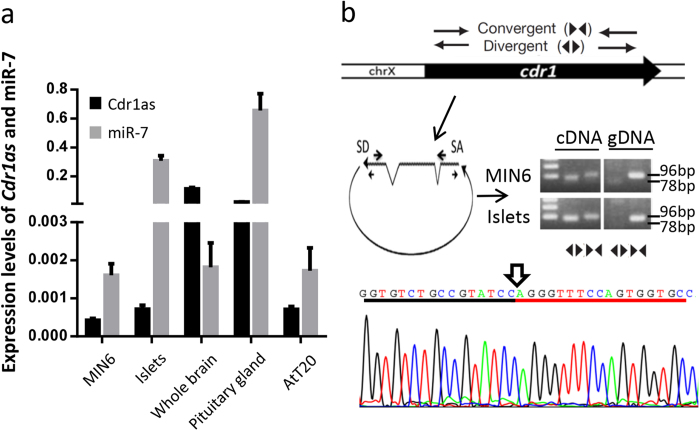
Characterization and functional analysis of *Cdr1as*. (**a**) *Cdr1as* and miR-7 expression profile in neuroendocrine tissues and cell lines. Expression levels of *Cdr1as* and miR-7 are normalized to *Gapdh* mRNA levels. Experiments were independently performed five times (*n* = 5) in triplicates. (**b**) Schematic illustration of the *Cdr1* locus with primers and validation of mouse *Cdr1as*. SD, splicing donor; SA, splicing acceptor. Sanger sequencing depicts the junction of *Cdr1as*.

The *Cdr1as* molecule was recently demonstrated to reduce miR-7 activity in embryonic zebrafish brain^6^,^7^. We hypothesized that *Cdr1as* may also affect miR-7 function in adult islet cells. Since miR-7 is abundantly expressed in islet cells, we assumed that *Cdr1as* is also expressed in islet cells and other neuroendocrine tissues. By using *Cdr1as* specific primers for qRT-PCR analysis, we confirmed the expression of *Cdr1as* in MIN6 cells and mouse islets. *Cdr1as* was also found to express in mouse brain, pituitary gland and AtT-20 cells (Fig. 1a). Concomitantly, the expression pattern of miR-7 was largely similar to that of *Cdr1as*, but its transcriptional level in islet or pituitary gland was much higher than *Cdr1as* (Fig. 1a).

Mouse *Cdr1as* is transcribed from the antisense strand of the *Cdr1* gene on chromosome X (NC_000086.7)^25^. We confirmed this event by using the divergent primers (*mus-ciRS-7* F and R), which amplified the predicted 78-bp of Cdr1as from mouse islet cDNA, but not genomic DNA, and also using the convergent primers (*mus-Cdr1* F and R), which amplified 96-bp of Cdr1 fragment from both cDNA and genomic DNA. The head-to-tail splicing junction of Cdr1as was further confirmed by Sanger sequencing (Fig. 1b).

### *Cdr1as* involving insulin signaling pathway

**Figure 2:**
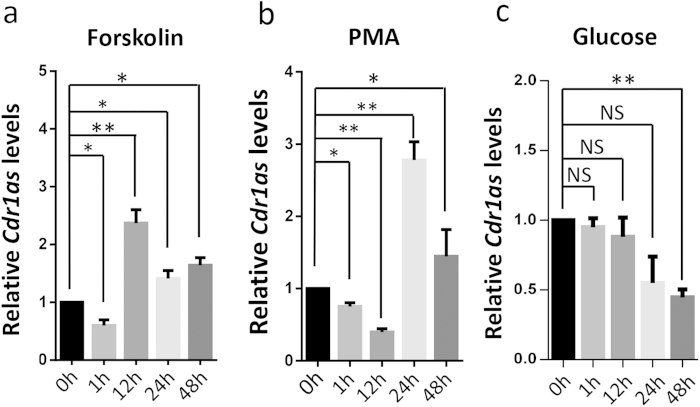
Effects of stimulators on *Cdr1as* expression. (**a**) *Cdr1as* expression in mouse islets treated by forskolin (10 μM). (**b**) PMA (1 μM) stimulated *Cdr1as* expression in mouse islets. (**c**) Effect of Glucose on *Cdr1as* expression in mouse islets. One-way ANOVA and Bonferroni test are used for statistical analysis. *n* = 3, *P < 0.05; **P < 0.01.

To learn whether *Cdr1as* is involved in insulin pathway in β cells, we measured *Cdr1as* expression levels in islet cells that commonly respond to several secretagogues such as forskolin, PMA and glucose. Following the stimulation at different time points (Fig. 2), we found that forskolin and PMA treatment dramatically increased *Cdr1as* transcriptional levels in mouse islets. In particular, by time-course analysis of forskolin treatment, *Cdr1as* levels in mouse islets were increased at longer-term, such as 2.6-fold at 12 h, 1.5-fold at 24 h, and 1.7-fold at 48 h, but not at short-term for 1 h treatment (Fig. 2a). These results suggest that *Cdr1as* expression could be induced by the forskolin-induced cAMP signal pathway. On the other hand, we observed ~2.8-fold and 1.5-fold increase of *Cdr1as* expression in mouse islets at 24 h and 48 h in response to the PMA treatment, following a significant decrease at 1 h and 12 h (Fig. 2b). These results showed that the PKC signaling pathway in islet cells, activated by PMA^26^,^27^, is also involved in the regulation of *Cdr1as* expression. However, after culturing with high glucose (16.7 mM), *Cdr1as* levels were decreased ~50% at 48 h, compared to basal glucose level at 3.3 mM in mouse islets (Fig. 2c). Similar results described in this section were also observed in MIN6 cells (data not shown).

### *Cdr1as* increased insulin secretion

Given the fact that miR-7 overexpression has been demonstrated to decrease insulin secretion and content in β cells^24^,^28^,^29^,^30^, it is possible that *Cdr1as* expression may affect insulin secretion via suppressing miR-7 activities. We first confirmed that the *Cdr1as* expression has direct inhibition on miR-7 activity in islet cells by measuring luciferase reporter activities with constructs either containing miR-7 binding site or the entire ciRS-7 sequence (Supplementary Fig. 1). Furthermore, after miR-7 or *Cdr1as* plasmid DNA was transfected into MIN6 cells and pancreatic islet cells for 48 h, miR-7 or *Cdr1as* expression was found to be increased ~70 folds or ~180 folds respectively, compared to the control (Supplementary Fig. 2). However, the endogenous miR-7 expression level, as measured by qRT-PCR, was found to be unaffected by exogenous transfection of the *pCDNA3-CiRS-7* (Supplementary Fig. 3a). Similarly, the endogenous *Cdr1as* expression level was not altered by exogenous transfection of the miRVec-miR-7 (Supplementary Fig. 3b). This observation confirmed that the inhibitory role of *Cdr1as* in miR-7 is associated with the binding of the target, not the degradation of miR-7^6^.

Since miR-7 interacts with *Cdr1as*, we examined whether endogenous miR-7 expression level is affected by forskolin, PMA and glucose treatment under the same condition as used in the stimulation of *Cdr1as* expression. Intriguingly, miR-7 expression was significantly decreased by these secretagogues (Supplementary Fig. 4), suggesting an unrecognized molecule or pathway that suppressed the expression of miR-7. This inhibitory effect on miR-7 expression, however, is beneficial for insulin secretion because miR-7 overexpression was found to reduce insulin secretion.

**Figure 3:**
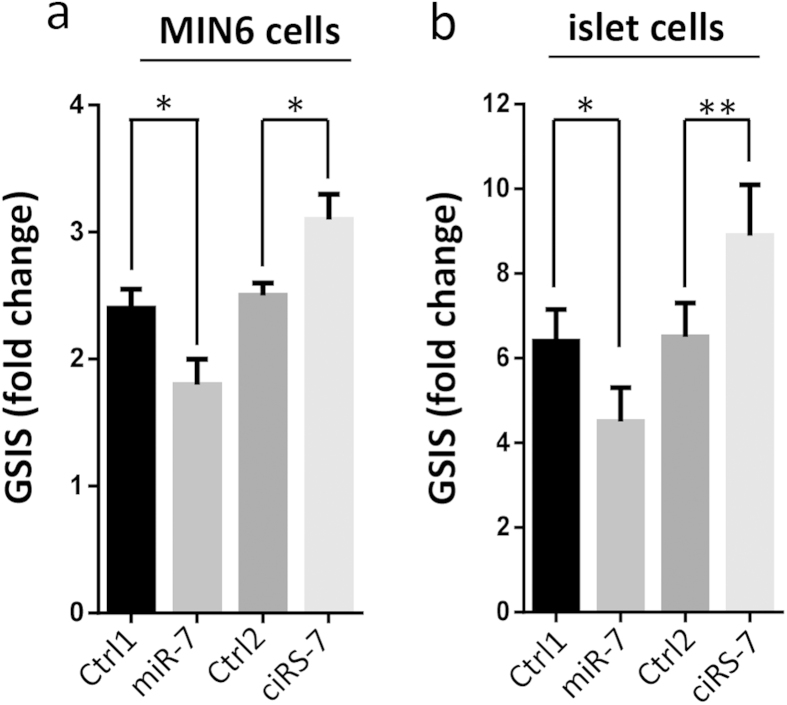
Impact of *Cdr1as* on insulin secretion. Insulin secretion is measured by GSIS assay in MIN6 cell (**a**) and mouse islet cells (**b**). *n* = 5, *P < 0.05, **P < 0.01.

Furthermore, we performed glucose-stimulated insulin secretion (GSIS) assay (see details in Materials and Methods) to evaluate the effects of overexpression of *Cdr1as* on insulin secretion. *Cdr1as* and miR-7 plasmid DNAs were separately transfected into MIN6 cells and also dissociated mouse islet cells in culture plates (see details in Materials and Methods). As expected, miR-7 overexpression reduced ~20% insulin secretion in MIN6 cells. In contrast, *Cdr1as* overexpression was found to increase ~30% of insulin secretion in MIN6 cells, compared to the control (Fig. 3a). Similar results were also observed in freshly isolated mouse islets, (Fig. 3b) i.e., ~30% decrease of insulin secretion in miR-7 overexpression group and ~40% increase in *Cdr1as* overexpression group (Fig. 3b).

### *Cdr1as* increased insulin content

**Figure 4:**
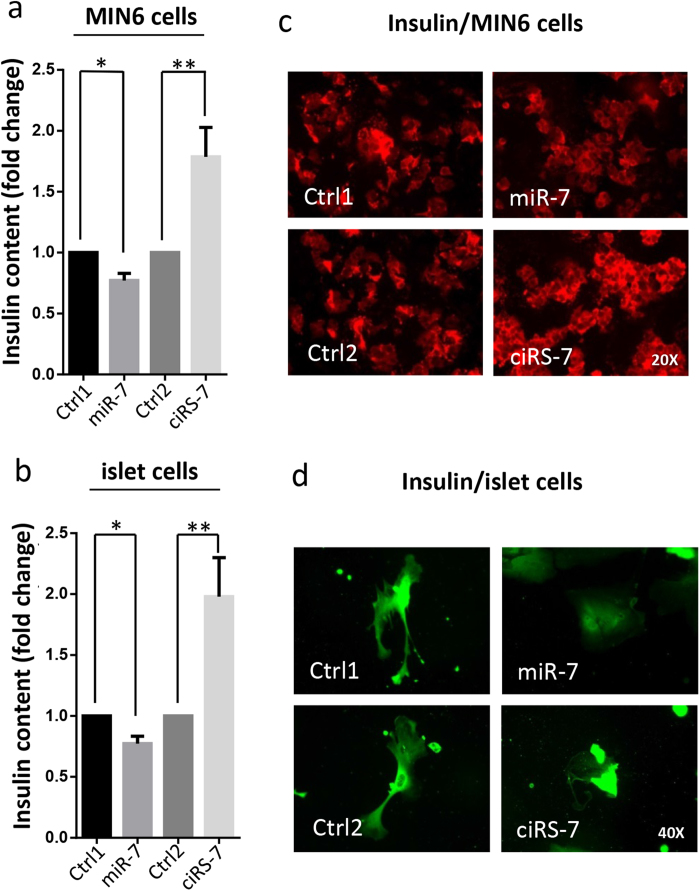
Insulin production is regulated by *Cdr1as*. (**a**) Insulin content in MIN6 cells measured by ELISA. (**b**) Insulin content in islet cells determined by ELISA. *n* = 3, *P < 0.05, **P < 0.01. (**c**) Insulin levels in MIN6 cells are visualized by immunostaining with insulin antibody. (d) Insulin levels in mouse islet cells are estimated by immunostaining with insulin antibody.

Meanwhile, we analyzed the effect of *Cdr1as* on insulin content. Using the insulin ELISA assay, we found that ~25% reduction of insulin content in miR-7 overexpressed MIN6 cells compared to a ~70% increase of insulin content in *Cdr1as* overexpressed MIN6 cells (Fig. 4a). In mouse islets cells, however, overexpression of ciRS-7 reached ~90% increase of insulin content compared to the control, while the miR-7 expression resulted in ~20% decrease of insulin content (Fig. 4b). Furthermore, the alterations of insulin contents were also visualized by immunofluorescence microscopy with antibodies to insulin. Decreased insulin content by miR-7 expression or increased insulin protein by *Cdr1as* expression was confirmed in MIN6 cells and islet cells (Fig. 4c,d). These results showed that *Cdr1as*, as a specific repressor of miR-7, is indeed implicated in the insulin pathway.

### Insulin transcripts were increased

**Figure 5:**
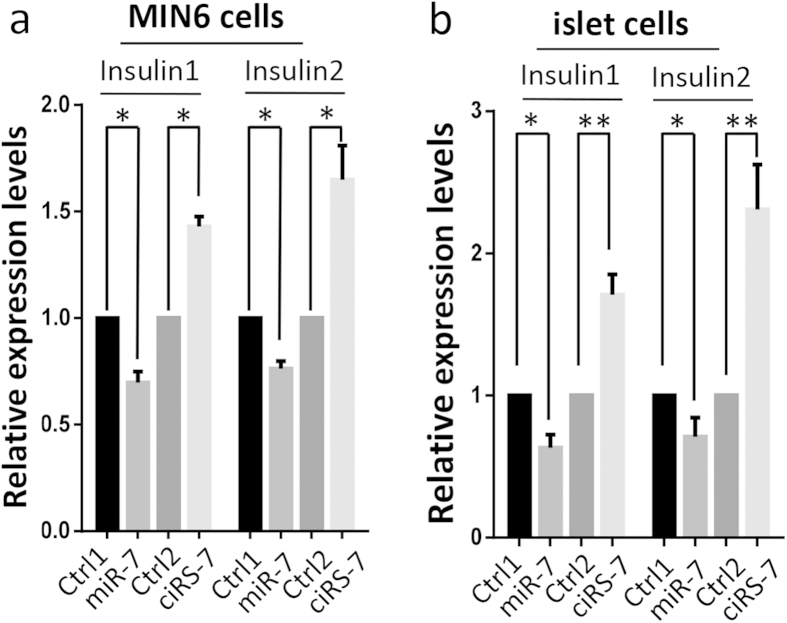
*Cdr1as* regulates insulin1 and insulin2 transcriptional levels. mRNA levels of insulin 1 and insulin 2 in MIN6 cells (**a**) or in mouse islets (**b**). *n* = 5, *P < 0.05, **P < 0.01.

Since insulin content was increased by the *Cdr1as* treatment, we examined whether overexpression of *Cdr1as* and/or miR-7 affects endogenous insulin1 and insulin2 mRNA levels. As determined by qRT-PCR, ~30% and ~20% reduction of insulin 1 and insulin 2 gene respectively were observed in the miR-7 overexpressed MIN6 cells (Fig. 5a). Similar results were observed in mouse islet cells (Fig. 5b). Conversely, we found 1.4-fold and 1.6-fold increase of insulin 1 and insulin 2 mRNA respectively in the *Cdr1as* overexpressed MIN6 cells (Fig. 5a). Likewise, we found 1.6-fold and 2.3-fold upregulation of insulin 1 and insulin 2 mRNA in *Cdr1as* overexpressed mouse islet cells (Fig. 5b). These results were consistent with the changes in insulin content (Fig. 4a,b) as well as the results of insulin immunostaining in MIN6 cells and islet cells (Fig. 4c,d). Altogether, our results indicate that the effect of *Cdr1as* on insulin content is through insulin biosynthesis, in which potential target genes of miR-7 may actually play an important role.

### *Myrip* and *Pax6* are miR-7 targets

To explore possible molecular interactions of *Cdr1as*/miR-7 in islet β cells, we analyzed potential miR-7 targets with two widely-used bioinformatics tools, PicTar (http://pictar.mdc-berlin.de) and miRNA.org (http://www.microrna.org/microrna/home.do). Among multiple hits of miR-7, two interesting genes were prioritized because of their role in insulin biosynthesis and exocytosis. Myrip (myosin VIIA and Rab interacting protein) is a partner of small GTPase Rab27 involving secretory granules transportation and release^31^,^32^. Also, Pax6 (paired box 6) is a transcriptional factor regulating insulin biosynthesis and secretion^33^,^34^, and a target of miR-7 reported previously^29^.

**Figure 6:**
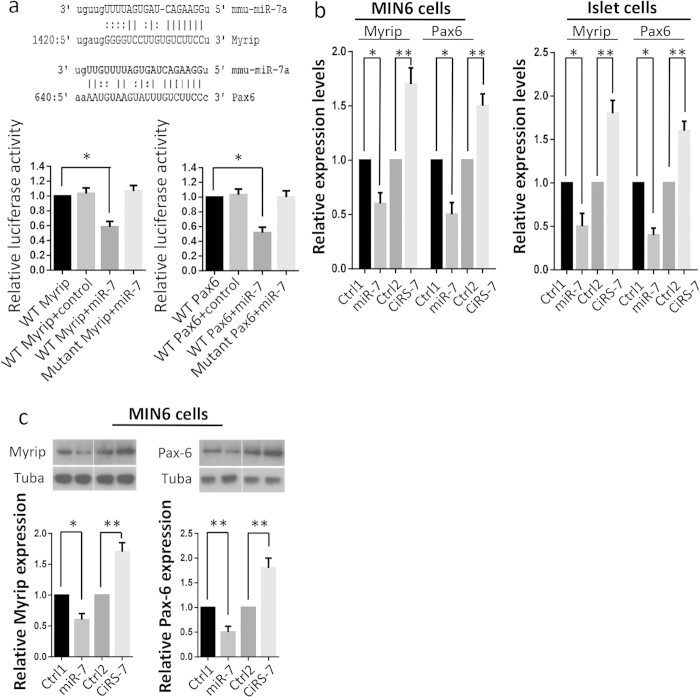
*Cdr1as* regulates insulin pathways by elevating effect on Myrip and Pax6. (**a**) The predicted miR-7a target sequence in the 3′-UTR sequence of wildtype (WT) or mutated (Mut) *Myrip* and *Pax6* (upper panel). After co-transfected with miR-7 in 293T cells, luciferase activities of wildtype 3′-UTR in *Myrip* or *Pax6* as well as mutated 3′-UTR in *Myrip* or *Pax6* are measured (lower panel). (**b**) Effects of miR-7 and *Cdr1as* overexpression on endogenous mRNA levels of *Myrip* or *Pax6* in MIN6 cells or islet cells. (**c**) Representative Western blots and quantitatively analysis show the effects of miR-7 and *Cdr1as* on the protein levels of Myrip and Pax6 in MIN6 cells. *n* = 3, *P < 0.05, **P < 0.01.

In order to demonstrate that *Myrip* is a novel direct target of miR-7, we established two dual-luciferase reporter constructs that contain either a wildtype or a mutated 3′-UTR of *Myrip*. In addition, we utilized wildtype or mutant 3′-UTR of Pax6 plasmids for target confirmation. Each of the construct DNAs was co-transfected with miR-7 expression vector or control vector into 293T cells. After 48 h culturing, luciferase activity of the cell lysates in each group was measured and compared. We observed that 40% reduction in the wildtype Myrip transfected cells and 50% reduction in Pax6 transfected cells when co-transfected with miR-7, but not co-transfected with control (Fig. 6a). However, luciferase activities in the mutant *Myrip* or the mutant *Pax6* did not show significant alterations because the mutations within the seed sequence of *Myrip* or *Pax6* abrogated the binding site of miR-7 (Fig. 6a). Thereby it was ascertained that miR-7 had an inhibitory effect on *Myrip* and *Pax6* through direct binding of their 3′-UTR. These results demonstrate that miR-7 could regulate the expression of *Myrip* and *Pax6*.

### *Cdr1as* upregulates miR-7 target genes expression

Furthermore, we examined whether *Cdr1as* and/or miR-7 are capable of modulating the expression of endogenous *Myrip* and *Pax6*. Forced expression of miR-7 in MIN6 cells was found to result in ~40% decrease of *Myrip* mRNA and 50% reduction of *Pax6* mRNA compared to the control plasmids-treated cells (Fig. 6b). While the forced expression of miR-7 in mouse islet cells showed even more decrease of *Myrip* mRNA (~50%) and *Pax6* mRNA (~60%) compared to their controls (Fig. 6b). In contrast, overexpression of *Cdr1as* significantly increased *Myrip* mRNA (~70%) and *Pax6* mRNA (~50%) in MIN6 cells (Fig. 6b). A similar but even better result was also observed in islet cells, i.e., ~80% increase in *Myrip* mRNA and ~60% in *Pax6* mRNA (Fig. 6b). A paralleled alteration was also confirmed at the translational level. Western blots confirmed that miR-7 reduced Myrip and Pax6 protein levels, while *Cdr1as* dramatically increased their expression in MIN6 cells (Fig. 6c). However, this finding was not repeated using mouse islet cells because of insufficient islet proteins for Western blotting.

## Discussion

**Figure 7:**
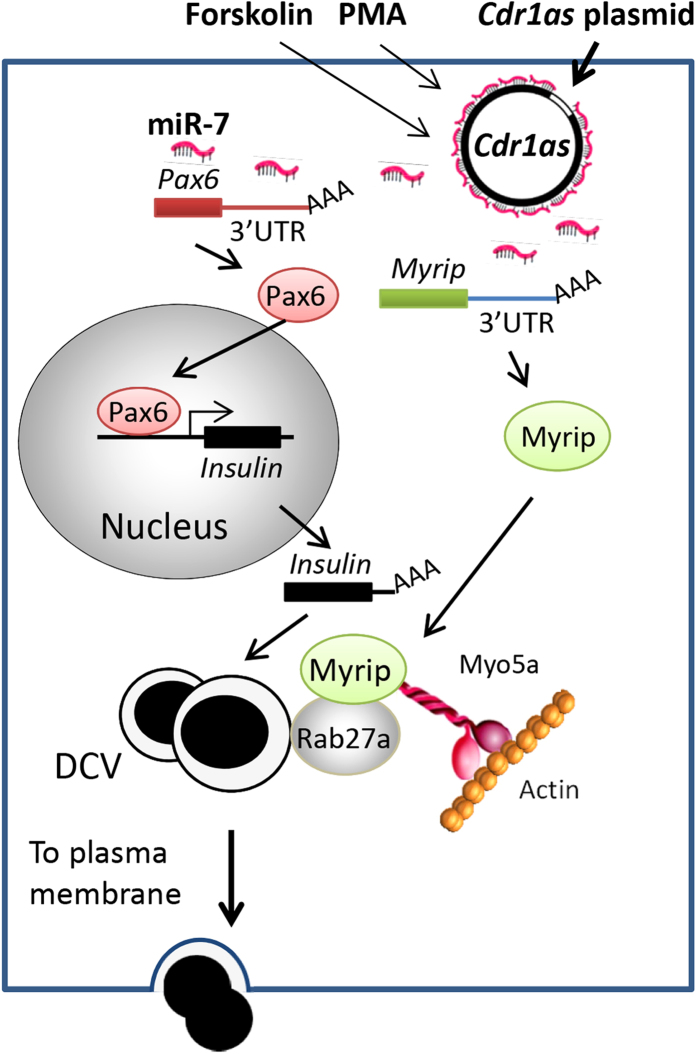
Working model of *Cdr1as*/miR-7-associated network in β cells. Overexpression of exogenous *Cdr1as* or stimulation of forskolin or PMA can significantly increase the expression level of *Cdr1as* in islet cells, which in turn inhibits miR-7’s function in insulin biosynthesis and secretion. In particular, physiological and biological functions of two major target genes (e.g., *Myrip* and *Pax6*) of miR-7 have been well studied in islet cells. The product of the *Myrip* gene was found to link the Rab27A-associated and insulin-containing granules (i.e., dense-core vesicle, DCV) via Myosin 5A to the actin filaments for DCV transportation towards plasma membrane and insulin secretion. On the other hand, the transcription factor Pax6 was found to enhance insulin transcription by binding to the promoters of insulin gene 1 and 2.

Widespread and substantial presence of circRNAs in the eukaryotic tree of life has been identified recently^10^. Initial characterization of circular RNAs and their underlying molecular mechanisms and potential application in islet cells need to be extensively investigated. In the present study, we showed for the first time that *Cdr1as* was expressed in islet cells, and its overexpression significantly increased insulin mRNA level and granule secretion in β cells. As shown in the working model (Fig. 7), we found that *Cdr1as* or miR-7 expression was upregulated or downregulated respectively by forskolin and PMA, indicating that *Cdr1as*/miR-7 is involved in the cAMP and PKC signal pathway. The opposite change thereby significantly increased the ratio of *Cdr1as* vs. miR-7 expression level in islet cells, suggesting most if not all of miR-7 will be inhibited by *Cdr1as*. Furthermore, we demonstrated that the effects of *Cdr1as* on insulin secretion are associated with the inhibition of miR-7 function, which also was shown in neuronal cells by previous studies^6^,^7^. Furthermore, we expanded the molecular interaction network of *Cdr1as*/miR-7 by adding one important target *Myrip* in islet cells (depicted in Fig. 7).

Although circRNAs-related research was occasionally reported in the past few decades, a large amount of circRNAs have been recently identified by high-throughput genome sequencing coupled with powerful computational analyses^5^,^6^,^7^,^8^,^9^,^10^,^11^. However, only a few of them like the *Cdr1as* have been functionally demonstrated to be miRNA sponges or inhibitors. In particular, *Cdr1as*, which is derived from an antisense transcript of the *CDR1* protein-coding gene at chromosome Xq27.1, contains 71 binding sites or 26 clusters corresponding to miR-7 sites. Among the human circRNAs, *Cdr1as* is the most compelling miRNA sponge for any conserved miRNA seed family identified so far^11^.

For the sake of strong effects on miR-7 function, *Cdr1as* could be an important regulator to prevent miR-7 from interacting with target transcripts in islet cells. The *miR-7* transcript has been detected to be the most abundant microRNAs in the human and mouse islets in terms of its highest ratio >150 between islet and surrounding acinar tissue while the ratio for *miR-375*, another well characterized microRNA for regulating insulin secretion in islets^35^, is less than 10^21^. As we mentioned earlier, transgenic mice overexpressing miR-7 in β cells developed diabetes due to impaired insulin secretion and β cell dedifferentiation. Importantly, genetic inactivation of miR-7 in β cells was found to result in increased insulin secretion but not affecting proliferation and apoptosis, indicating that miR-7 is dispensable for the maintenance of endocrine β cell mass^24^. In contrast, deletion of miR-375 in islets resulted in moderate hyperglycemia^35^. Furthermore, an interesting observation is that inactivation of miR-7 in obese mice might be sufficient to rescue β cell failure and glycemia^24^. Therefore, it is of importance to explore the function of *Cdr1as* in islet pathophysiology and treatment of obese diabetics.

Overexpression of *Cdr1as* in islet cells was predicted to result in alterations of insulin secretion because of the function of miR-7 as we discussed above. Under current experimental condition, we indeed observed significantly increased insulin secretion and content in MIN6 and mouse islet cells. Further studies of *Cdr1as* in transgenic mouse model may disclose its effects on the insulin pathway in obese diabetic mice. The underlying mechanism of how *Cdr1as*/miR-7 improved β cells function certainly requires further investigations.

As we mentioned earlier, endogenous *Cdr1as* expression was increased by forskolin and PMA, but not glucose, suggestive of the involvement of cAMP and PKC pathways. MiR-7 expression also responded in a similar way to these secretagogues. Whether these responses are derived from associated promoter elements like CREB binding sites or from indirect elements of the *Cdr1as*/miR-7 network remains to be studied. Nevertheless, further studies are required to elucidate the molecular mechanism of how *Cdr1as* expression is regulated in islet cells. Also, the identification of miR-7 target genes related to insulin signaling pathway in islet β cells may reveal the molecular network responsible for the insulin secretion and homeostasis.

Although many miR-7 target genes were predicted by miRNA software, only a dozen of them have been experimentally demonstrated to be direct targets in insulin pathway of adult islet β cells. Examples include the regulators in granule exocytosis by SNARE (*Snca*, *Cspa*, and *Cplx1*), calcium-response elements (*Pkcb*), cytoskeleton components (*Pfn2*, *Wipf2*, *Basp1*, and *Phactr1*), and transcriptional factor (*Pax6*)^24^,^29^. In fact, the increased insulin secretion was shown in both acute phase and second phase in the miR-7 deletion mouse^24^. Few of these targets like *Snca* were found to be implicated in acute phase of insulin release. However, more evidences are needed to elucidate how most of these target genes are involved in insulin pathway in islets. Apparently, the inactivation of miR-7 in mouse islets showed broad effects on insulin pathway due to alterations of hundreds of potential targets. Therefore, additional miR-7 targets involving insulin granule metabolism as well as insulin homeostasis should be identified.

Since we observed increased insulin mRNAs in the *Cdr1as* overexpressed islet cells, elevated Pax-6 expression levels was detected in responding to the *Cdr1as* expression (Fig. 7). This finding is in agreement with the inhibitory role of miR-7 on Pax-6 by ours and others^29^. As a homeobox-containing transcriptional factor, Pax6 enhances insulin gene 1 (*Ins1*) and gene 2 (*Ins2*) transcripts by directly binding to their promoters, which ultimately increased insulin content and secretion. Conversely, knockdown of Pax6 in islet cells resulted in impairment of glucose-induced insulin secretion and decrease in insulin content in primary β cells^29^,^34^. Furthermore, conditional knockout Pax6 in mouse pancreas showed diabetic phenotypes and reduced number of insulin-expressing cells^36^. These findings clearly showed that Pax-6 levels, as a major target of miR-7, is likely to be upregulated by *Cdr1as* in islet cells.

To identify new targets of miR-7 in insulin secretion pathway, we screened hundreds of candidate genes that were predicted by multiple bioinformatic tools and extensively analyzed the candidate’s function in islet cells. After measuring 3′-UTR luciferase reporter activity of the selected candidate gene, we further confirmed that *Myrip* expression level was decreased by miR-7 but increased by *Cdr1as* in the islet cells (Fig. 7). The function of Myrip in regulation of insulin exocytosis has been documented because it forms tripartite complex with Rab27a and MyosinVa to mediate insulin granule transportation and secretion^31^,^37^,^38^. Interestingly, this interaction between Myrip and MyosinVa was activated by cAMP pathway^39^, which is inversely correlated with miR-7 by forskolin treatment observed in this study. In fact, miR-7 expression was reduced in either glucose or forskolin, whereas *Cdr1as* expression showed positive response to both secretagogues. In addition, the interaction of *Cdr1as* with miR-7 could be targeted by another miRNA, miR-671, which triggers endonucleolytic cleavage of *Cdr1as*^25^. These results revealed the complex network in islet cells for regulation of insulin homeostasis.

Taken together, our results demonstrated a potential application of *Cdr1as* on improving β cell function, and also provided a new insight into circRNA regulatory network in islet cells. *Cdr1as* may represent a useful tool in addressing the growing request of new therapeutic strategies based upon insulin secretion and β cells renewal in diabetes. In this regard, the pathophysiological role of *Cdr1as* in islet cells requires further investigations.

## Materials and Methods

### Cell lines

MIN6 cells (passage 65) were cultured in DMEM medium supplemented with 15% FBS as previously described^40^. 293T cells were cultured in DMEM medium supplemented with 5% FBS. All cell lines were cultured at 37 °C in 5% CO_2_.

### Plasmids

The method of construction of the wide-type (WT) and mutant Myrip luciferase reporter was previously reported^41^. A 673-bp fragment of 3′-UTR of Myrip was amplified from MIN6 cells cDNA and cloned into *XhoI* and *NotI* sites of the luciferase reporter pmirGLO vector (Promega, Madison WI) with primers (WT-Myrip). Myrip 3′-UTR mutant seed sequence was amplified with four overlapping PCR primers (WT-Myrip and Mut-Myrip). Primer sequences are listed in the Supplementary Table 1. WT and mutant type of luciferase reporter constructs were validated by DNA sequencing. PmirGlo-Pax6 WT and pmirGlo-Pax6 mut plasmid were gifts from Dr. N. Coré (Aix-Marseille University, France). MiR-7 (miR-7a is dominantly expressed in islets) expression plasmid, miRVec-miR-7 and its control plasmid (harboring a scrambled sequence, named as “Ctrl1”) were kindly provided by Dr. R. Agami (Netherlands Cancer Institute, Netherlands). Four plasmids, including *Cdr1as* expression plasmid, pCDNA3-ciRS-7 and its scrambled sequence plasmid (which inserted a *Cdr1as* sequence only but no invert repeat flanking introns, resulting in no circular *Cdr1as* production; named as “Ctrl2”), psiCheck-miR-7 and psiCheck-CiRS-7, were kindly given by Dr. T. Hansen (Aarhus University, Denmark).

### Mouse islets isolation and dissociation

Islets were isolated from 3-4 month-old and sex-matched C57BL/6 mice with Collagenase P Solution (1 mg/ml, Roche, Indianapolis, IN) as previously described^42^. Briefly, Collagenase P solution was injected into the mouse common bile duct to inflate the pancreas followed by removal and incubation of inflated pancreas at 37 °C for digestion until the pancreas to form a milky solution with only a few clumps. After digestion, islets were purified in density gradient Histopaque1077, 1083, and 1119 (Sigma, St. Louis, MO). Finally, islets were manually selected and washed with Krebs-Ringer HEPES (KRBH) buffer, and cultured overnight in RPMI-1640 full medium (supplemented with 15% FBS, 100 U/ml penicillin, 0.1 mg/ml streptomycin, and 5 mM glucose). About 200 islets were dissociated in the dissociation solution (HBSS with 1% BSA, 3 mM EGTA and 0.025% trypsin, PH 7.4) per group. Single islet cells were then selected and plated as a monolayer onto poly-L-lysine-coated six-well dishes. Cells were used on the 2nd day after dispersion. Islet cells were cultured in RPMI-1640 full medium with 5 mM glucose at 37 °C and further processed at 48 h after plasmid DNA transfection. Mice in this study were breeder and maintained following with the recommendations in the Guide for the Care and Use of Laboratory Animals of the National Institutes of Health, USA. All animal care and experiments were performed in accordance with the guidelines and were approved by the Ethics Committee of College of Wenzhou Medical University.

### Total RNA, miRNA isolation, and quantitative PCR

Total RNAs and miRNAs were extracted from cultured cells and C57BL/6 mouse tissues using miRNeasy Mini kit (Qiagen, Valencia, CA). Quantitative RT-PCR for measuring mRNA and miRNA levels was performed using miScript II RT kit and miScript SYBR Green PCR kit (Qiagen) in a 7500 Real-Time PCR system (Applied Biosystems). Primer sequences are also provided (Supplementary Table 1).

### Insulin secretion and content in MIN6 cells and mouse islets

MIN6 cells and dissociated islet cells were transfected with miRVec-miR-7, pCDNA3-ciRS-7 and their control plasmids respectively using Lipofectamine 2000 (Invitrogen, Grand Island, NY) for further glucose stimulated insulin secretion (GSIS) assay. The transfection efficiency of the MIN6 and dissociated islet cells were about 30% and 25%, respectively, as evaluated by cotransfected EGFP-expressing plasmid. After 48 h incubation, these cells were washed twice in buffer A (5 mM KCl, 120 mM NaCl, 24 mM NaHCO3, 1 Mm MgCl2, 2 mM CaCl2, 1 mg/ml Ovalbumin, 15 mM HEPES at pH 7.4) and then transferred to buffer A with 3.3 mM glucose for 2 hours to stabilize basal insulin secretion. After MIN6 and Islet cells were rinsed twice with buffer A without glucose, glucose stimulation was performed in buffer A with either 3.3 mM glucose or 16.7 mM glucose for 1 hour. Insulin levels in culture medium and in cell lyses were measured with Mouse High Range Insulin ELISA kit (ALPCO Diagnostics, Salem, NH). Fold changes were calculated between the basal insulin and the stimulated insulin level.

### Immunofluorescence

Transfected MIN6 and islet cells in chamber slides were fixed by 4% paraformaldehyde and stained with primary rabbit polyclonal insulin antibody (1:500 dilution, Santa Cruz Biotech) or a mouse monoclonal insulin antibody (1:500 dilution, Sigma) and secondary antibodies Alexa Fluor 568 Goat Anti-Rabbit IgG (H+L) Antibody or Alexa Fluor 488 Goat Anti-Mouse IgG (H+L) Antibody (1:1000 dilution, Invitrogen). Vectashield mounting medium containing DAPI (Vector Laboratories, Burlingame, CA) was used for nuclear staining. Images were captured with ZEN Imaging Software (ZEISS, Thornwood, NY) using constant exposure parameters for each fluorescence channel.

### Cell transfection and luciferase analysis

293T cells were co-transfected with *Myrip* or *Pax6* 3′-UTR luciferase reporter plasmid DNA and miRVec-miR-7 or its control plasmid DNAs at 1:10 ratio and were then harvested after 48 h in culture. Islets cells were transfected with plasmid Ctrl2 or pcDNA3-ciRS-7, together with or without miRVec-miR-7 and psiCheck reporter plasmids. The levels of Renilla luminescence were normalized to the internal control firefly luminescence. All transfections use HiPerFect Transfection Reagent (Qiagen) according to the manufacturer’s instructions. Luciferase activities were detected using the Dual-Luciferase Reporter Assay System (Promega).

### Protein extraction and Western blot analysis

Protein was isolated from cell lysis using Mammalian Protein Extraction Reagent (Thermo Fisher Scientific, Rockville, MD). Equivalent amount of protein was loaded on Novex Bis-Tris PAGE gels (Invitrogen), and transferred and blocked in blocking buffer (TBS/0.1% Tween20 (TBS-T)/5% nonfat-milk). After blocking for 2 hour, blots were incubated over night at 4 °C with anti-Myrip antibody (1:500 dilution, Santa Cruz Biotechnology, Santa Cruz, CA), Anti-Pax6 antibody (1:1000 dilution, Santa Cruz Biotechnology), or α-tubulin (1:5000 dilution, Abcam, Cambridge, Massachusetts). The blots were subsequently processed using the ECL Western Blotting Detection Reagent (GE Healthcare, Laurel, MD) and quantitated using NIH ImageJ.

### Statistical analysis

Each experiment was performed in triplicates. All data were represented in mean ± SEM. Student *t* test and one-way ANOVA and Bonferroni test were used to determine statistical significance. p < 0.05 was considered to represent a significant difference.

## Additional Information

**How to cite this article**: Xu, H. *et al.* The circular RNA *Cdr1as*, via miR-7 and its targets, regulates insulin transcription and secretion in islet cells. *Sci. Rep.* **5**, 12453; doi: 10.1038/srep12453 (2015).

## Supplementary Material

Supplementary Information
