# Supplementary material for: The circular RNA Cdr1as, via miR-7 and its targets, regulates insulin transcription and secretion in islet cells
Source: Sci Rep. 2015 Jul 27;5:12453. doi: 10.1038/srep12453 (PMC4515639; doi:10.1038/srep12453)
Supplement: Supplementary Information [file srep12453-s1.pdf]

# Supplementary information files

## The circular RNA *Cdr1as*, via miR-7 and its targets, regulates insulin transcription and secretion in islet cells

Huanyu Xu, [1]* Sen Guo, [1] Wei Li, [2] Ping Yu, [1]*

[1]Institute of Genomic Medicine, Wenzhou Medical University, Zhejiang, China, [2]Zhejiang Provincial Key Laboratory of Medical Genetics; School of Laboratory Medicine & Life Sciences, Wenzhou Medical University, Zhejiang, China

**Corresponding author**

Ping Yu, and Huanyu Xu;

Institute of Genomic Medicine, Wenzhou Medical University, Zhejiang province, China;

Phone: +86 159-8878-1270; Fax: +86-577-86699399

E-mail: jyzwzyu@gmail.com and xuhuanyu1979@gmail.com

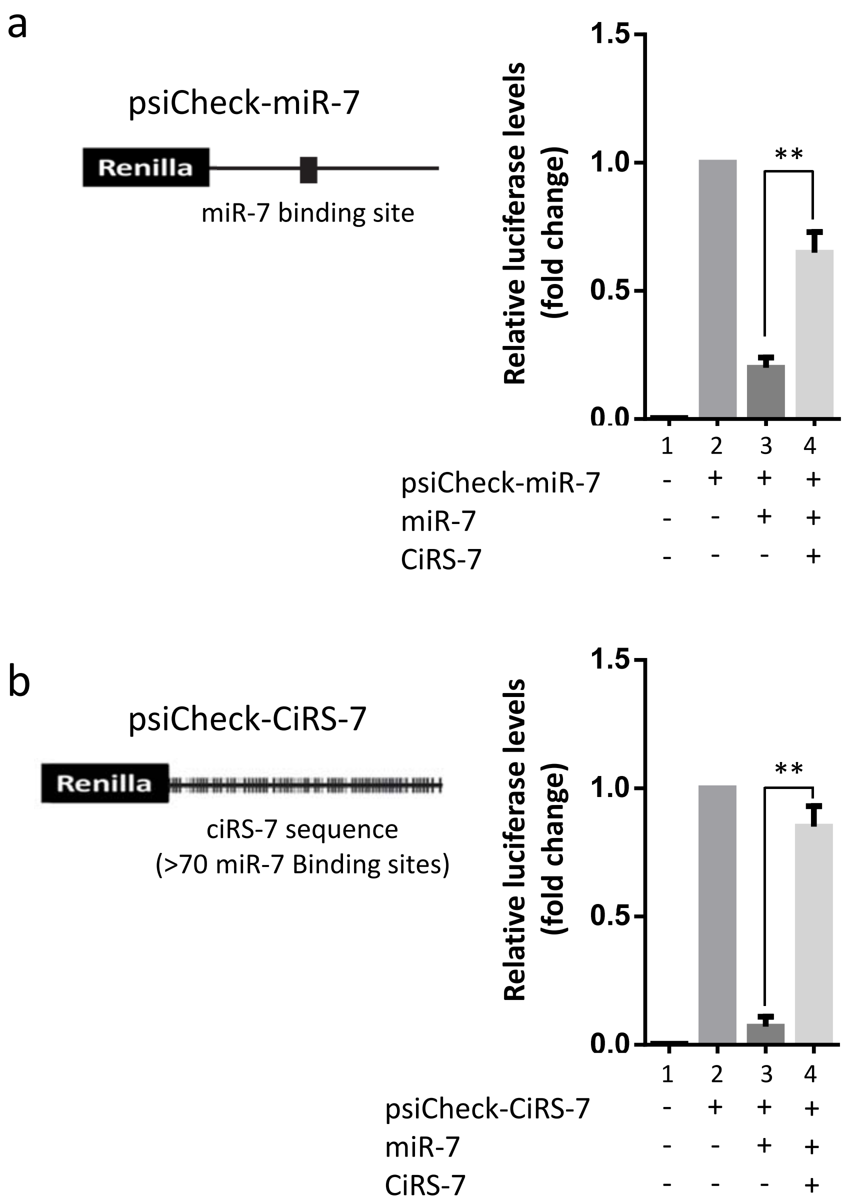


**Supplementary Fig. 1** Physical interaction of Cdr1as and miR-7 in islet cells. (**a**) The renilla activity of the psiCheck-miR-7 (column 2) was suppressed about 80% by transfection of miR-7 (column 3), but not scrambled miR-7 (not shown). However, transfection of *Cdr1as* (column 4) significantly rescued the inhibitory effect of miR-7 on renilla activity. (**b**) Likewise, the renilla activity of the psiCheck-CiRS-7 (column 2) was also suppressed about 90% by transfection of miR-7 (column 3), but not scrambled miR-7 (not shown). However, transfection of *Cdr1as* (column 4) significantly rescued the inhibitory effect of miR-7 on renilla activity. Luciferase activity, as the corresponding gene is included in the same vector, was served as an inner control and measured by using a Microplate Luminometer (Berthold Technologies, Germany). $n$ =3, Mean $\pm$ SEM, representing three independent experiments In triplicates. **, $P < 0.01$

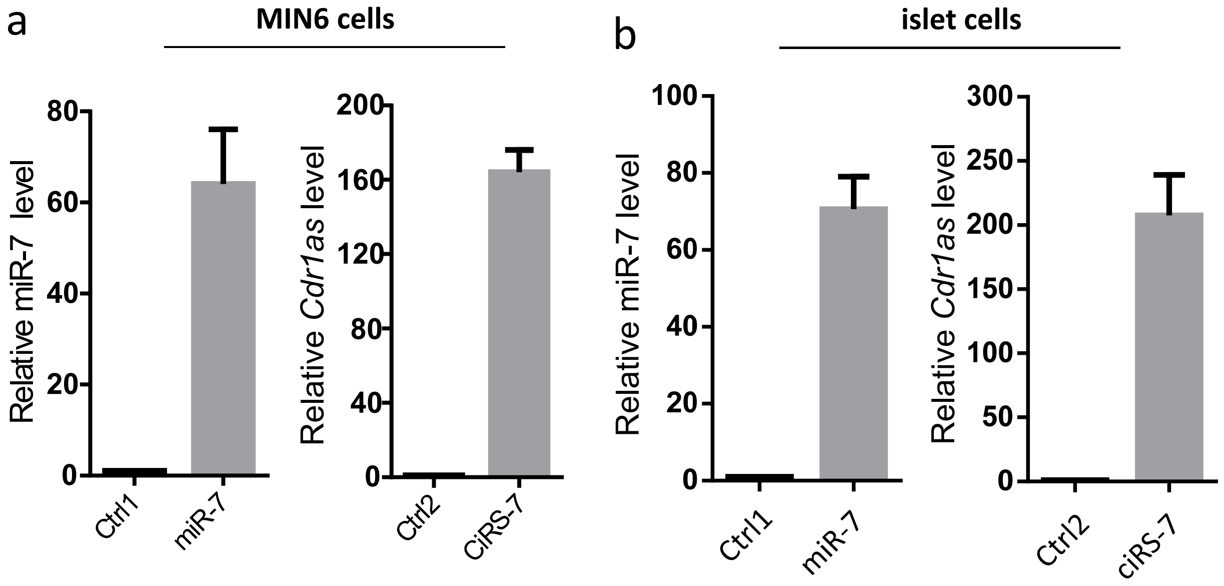


**Supplementary Fig. 2** – Validation of expression miR-7 and *Cdr1as* expression in MIN6 cells (**a**) and islet cells (**b**) by transfecting each corresponding plasmid (miRVec-miR-7 or pCDNA3-ciRS-7) for 48, and compared with controls (see details in Materials and Methods). Experiments were independently performed for three times in triplicates.

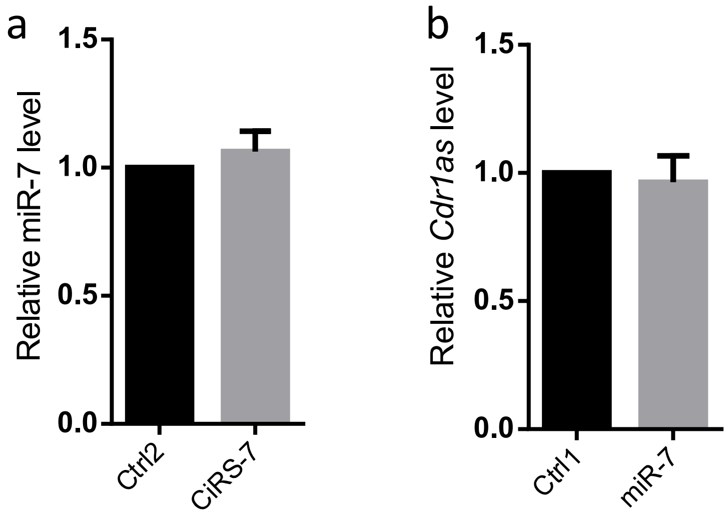


**Supplementary Fig. 3** Quantification of miR-7 (**a**) or *Cdr1as* level (**b**) levels derived from transfections with pCDNA3-ciRS-7 along with plasmids expressing miR-7 (miRVec-miR-7). Experiments were independently performed three time in triplicates.

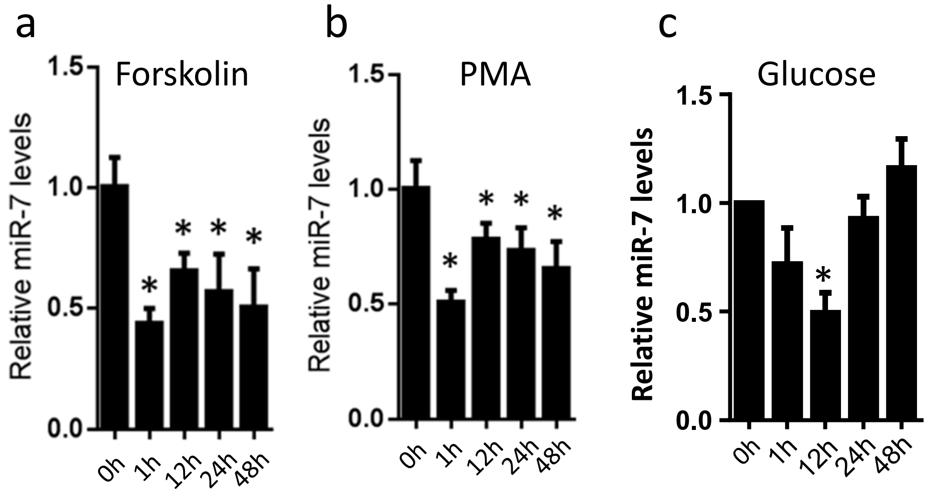


**Supplementary Fig. 4** miR-7 expression responding to forskolin, PMA and glucose. (**a**) miR-7 expression in islets treated by forskolin (10 µM). (**b**) PMA (1 µM) stimulated miR-7 expression in islets. (**c**) Effect of glucose on miR-7 expression in islets. *n* = 3, *, *P* < 0.05; **, *P* < 0.01.

Supplementary Table 1. Primers used for quantitative real-time PCR

| Primer name | Forward primer (5'-3') | Reverse primer (5'-3') |
|---|---|---|
| miR-7 | TGGAAGACTAGTGATTTTGTTGT | Universal primer(Qiagen) |
| mus-ciRS-7 | GTGTCTCCAGTGTATCGGCG | TACTGGCACCACTGGAAACC |
| Mus-cdr1 | TGGAAGATCACGATTGTCTGGA | GGTGCCAGTACCAAGGTCTTC |
| Pax6 | GCGGAGTTATGATACCTACACC | GAAATGAGTCCTGTTGAAGTGG |
| WT-Myrip | GCaagcttCAGGGCAGGGGCTGAAATAA | ACACTGGTAGCACAAAGGCAtgatcaGC |
| Mut-Myrip | TGTGaCaagCTATGATGCTCCT | ATAGcttGtCACAAGGACCCCC |
| Q-Myrip | TCGAAGCATCTTCCGTGACC | GGTCAAGGCACTGTCGTGTA |
| GAPDH | GTCGGTGTGAACGGATTTG | GAATTTGCCGTGAGTGGAG |
| Insulin1 | AAGCAGGTCATTGTTTCAACA | TTGGGTGTGTAGAAGAAGCC |
| Insulin2 | GCGTGGCTTCTTCTACACACC | CCAGTGCCAAGGTCTGAAGG |
